# Lensfree Fluorescent On-Chip Imaging of Transgenic *Caenorhabditis elegans* Over an Ultra-Wide Field-of-View

**DOI:** 10.1371/journal.pone.0015955

**Published:** 2011-01-06

**Authors:** Ahmet F. Coskun, Ikbal Sencan, Ting-Wei Su, Aydogan Ozcan

**Affiliations:** 1 Electrical Engineering Department, University of California Los Angeles, Los Angeles, California, United States of America; 2 California NanoSystems Institute (CNSI), Los Angeles, California, United States of America; Massachusetts General Hospital, United States of America

## Abstract

We demonstrate lensfree on-chip fluorescent imaging of transgenic *Caenorhabditis elegans* (*C. elegans*) over an ultra-wide field-of-view (FOV) of e.g., >2–8 cm^2^ with a spatial resolution of ∼10µm. This is the first time that a lensfree on-chip platform has successfully imaged fluorescent *C. elegans* samples. In our wide-field lensfree imaging platform, the transgenic samples are excited using a prism interface from the side, where the pump light is rejected through total internal reflection occurring at the bottom facet of the substrate. The emitted fluorescent signal from *C. elegans* samples is then recorded on a large area opto-electronic sensor-array over an FOV of e.g., >2–8 cm^2^, without the use of any lenses, thin-film interference filters or mechanical scanners. Because fluorescent emission rapidly diverges, such lensfree fluorescent images recorded on a chip look blurred due to broad point-spread-function of our platform. To combat this resolution challenge, we use a compressive sampling algorithm to uniquely decode the recorded lensfree fluorescent patterns into higher resolution images, demonstrating ∼10 µm resolution. We tested the efficacy of this compressive decoding approach with different types of opto-electronic sensors to achieve a similar resolution level, independent of the imaging chip. We further demonstrate that this wide FOV lensfree fluorescent imaging platform can also perform sequential bright-field imaging of the same samples using partially-coherent lensfree digital in-line holography that is coupled from the top facet of the same prism used in fluorescent excitation. This unique combination permits ultra-wide field dual-mode imaging of *C. elegans* on a chip which could especially provide a useful tool for high-throughput screening applications in biomedical research.

## Introduction

*C. elegans* is an important model organism that has been widely studied in various fields such as genetics [1], oncology [2] and neurobiology [3]. Wide-field optical imaging of *C. elegans* is an essential need for all these fields to enable high-throughput screening of this model organism. While several high-throughput imaging platforms have been successfully demonstrated so far [4]–[13], the main stream for this application involves the use of lens-based conventional optical microscopes which can only provide a limited field-of-view (FOV) of e.g., ≤1mm^2^, and therefore require mechanical scanning to provide a larger imaging FOV. In addition to this, such conventional optical microscopy platforms are rather bulky, and do not provide a decent match in terms of compactness to micro-fluidic technologies that are becoming widely used today in high-throughput screening of *C. elegans*.

**Figure 1:**
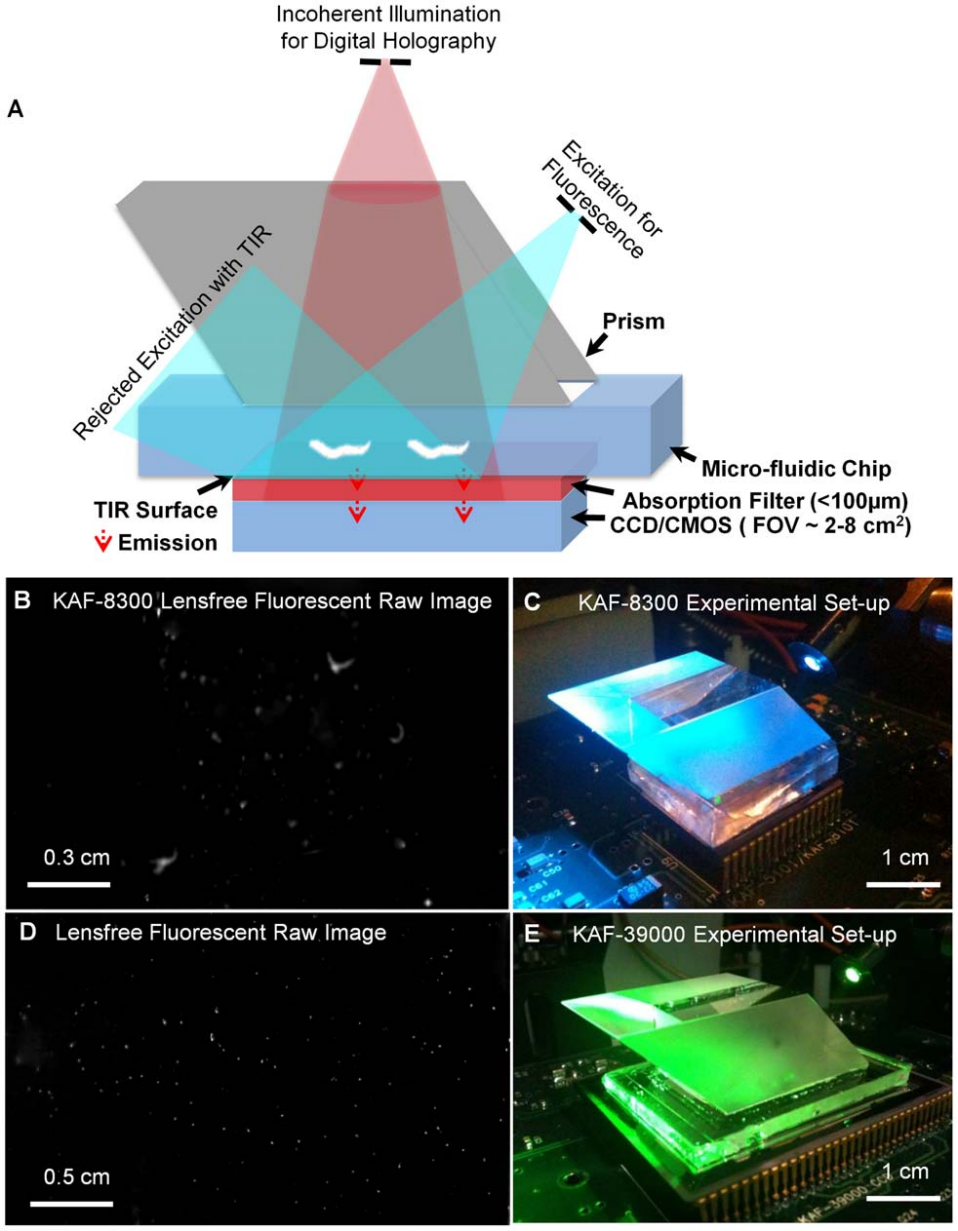
Lensfree on-chip fluorescent and holographic imaging platform. (a) Schematic diagram of lensfree on-chip fluorescent and holographic imaging platform is shown. Fluorescent excitation is achieved through the side facet of a rhomboid prism using an incoherent source (i.e. spectrally filtered Xenon lamp); and holographic illumination is achieved through the top facet of the same prism using an LED (632 nm peak, and ∼20 nm bandwidth). (b) Lensfree fluorescent imaging is demonstrated over >2 cm^2^ using KAF-8300 sensor (Full Frame CCD with a pixel size of 5.4 µm). (c) shows the experimental set-up of lensfree fluorescent imaging platform for the same sensor. The fluorescent *C. elegans* samples were excited through a prism interface, where an index matching oil was used to assemble the chip and the prism. Only the fluorescent emission emerging from gene-expressed parts of the worm body is detected by the KAF-8300 sensor-chip. (d) On-chip lensfree fluorescent imaging is illustrated over >8cm^2^ using KAF-39000 sensor (Full Frame CCD with a pixel size of 6.8 µm). (e) shows the experimental set-up of lensfree fluorescent imaging platform for the same sensor. Note that the excitation source in both of these set-ups is tunable, and therefore various fluorescent dyes could potentially be used without an issue.

To provide an alternative solution to this imaging need, here we demonstrate ultra-wide field fluorescent imaging of transgenic *C. elegans* over an FOV of >2–8 cm^2^ with a spatial resolution of ∼10 µm. This platform relies on lensfree on-chip imaging which, broadly defined, is becoming an important substitute for conventional microscopes especially for high-throughput imaging applications [14]–[21]. In our on-chip fluorescent imaging platform (see Fig. 1), transgenic *C. elegans* samples are excited through a prism interface where the excitation light is rejected based on total internal reflection (TIR) that is occurring at the bottom facet of the glass substrate. The emitted fluorescent signal from the body of the worm does not entirely obey TIR and therefore can be detected by a wide-field opto-electronic sensor-array e.g., a charge-coupled-device (CCD) without the use of any lenses. This detection process occurs through a wide angular range (corresponding to numerical aperture of ∼1.0) and because the fluorescent emission from the sample is not directional, the point-spread-function of such a lensfree fluorescent imaging platform will be rather broad, which will significantly limit the achievable raw spatial resolution. To combat this problem we have recently demonstrated the use of a deconvolution approach (namely the Lucy-Richardson algorithm [22]–[24]) to achieve ∼40–50µm resolution in lensfree fluorescent imaging of e.g., labeled white blood cells [14]. While this is still a useful resolution level for on-chip detection and counting of e.g., rare cells, a significant performance improvement would be needed to lensfree image *C elegans* samples with much better resolution.

For this end, in this manuscript we demonstrate the use of a more appropriate reconstruction algorithm for lensfree on-chip imaging of transgenic *C. elegans* samples to achieve a significantly improved resolution of ∼10 µm over a wide FOV. This approach is based on compressive sampling theory [25]–[27], which aims to recover a sparse function from much fewer samples than it would be required according to Shannon's sampling theorem. Transgenic *C. elegans* samples by definition satisfy the sparsity constraint of compressive sampling, and therefore can be efficiently decoded by using various compressive decoders that have been developed recently [28]–[30]. ***Our results constitute the first time that a lensfree on-chip platform has successfully imaged fluorescent C. elegans samples.***

In addition to wide-field fluorescent imaging, we also demonstrate that the same lensfree on-chip platform can also conduct bright-field transmission imaging of *C. elegans* samples using partially coherent digital in-line holography [18], [19], which is coupled to the same platform through the top facet of the prism as illustrated in Fig. 1. We believe that such ultra-wide field dual-mode imaging of *C. elegans* on a chip might provide a useful high-throughput tool for biomedical research in various fields including genetics, oncology and neurobiology.

## Results and Discussion

Initially, to test the performance of our lensfree on-chip imaging platform, we imaged fluorescent micro-beads (4 µm diameter, Excitation: 505nm, Emission: 515nm) by using two different sensor chips as illustrated in Fig. 1. In specific, we worked with two full-frame CCD chips namely, KODAK KAF-8300 (5.4 µm pixel size, ∼2.4 cm^2^ active imaging area) and KODAK KAF-39000 (6.8 µm pixel size, ∼18 cm^2^ active imaging area). In our lensfree fluorescent imaging modality, because the fluorescent detection occurs at extremely oblique angles on the sensor chip, depending on the opto-electronic design of the pixels and the underlying circuitry of a given chip, the fluorescent point-spread function (PSF) of our platform would exhibit a noticeable variance in its 2D pattern from one sensor-chip to another, which requires calibration of each chip by measuring its unique PSF. Therefore the main purpose of using different sensor chips in this work was to demonstrate sensor independent performance of our lensfree imaging modality.

**Figure 2:**
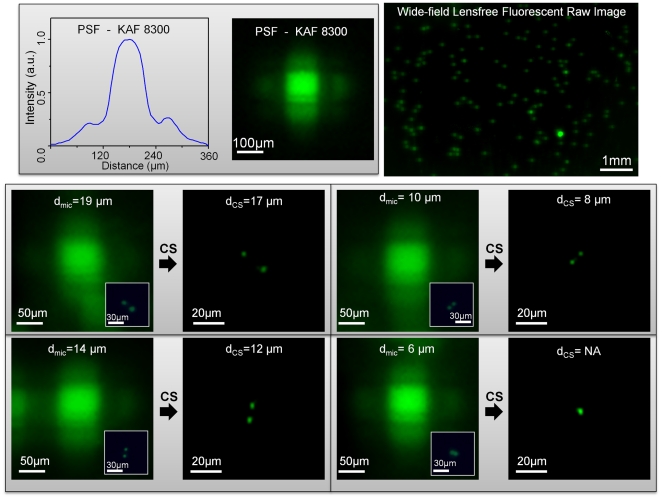
Lensfree fluorescent images of various 4 µm bead-pairs. (Top Row) illustrates wide-field lensfree fluorescent imaging results for 4 µm fluorescent beads recorded using KAF-8300 sensor. The *measured* point-spread-function (PSF) of the same system (corresponding to 4 µm diameter fluorescent beads) is also shown at the top left. The 2D pattern of this PSF presents a *unique* signature, which is mostly dictated by the opto-electronic design of the CCD chip. (Middle and Bottom Rows) Lensfree fluorescent images of various 4 µm bead-pairs are shown. For comparison purposes, the inset images in each frame also show conventional fluorescent microscope images of the same closely-packed beads. Based on these microscope images, the center-to-center distances (**d_mic_**) between the fluorescent particles are calculated. Using compressive sampling (CS - as indicated with the solid black arrows), lensfree fluorescent raw images are decoded to resolve the individual fluorescent particles from each other. These decoding results nicely match to the corresponding microscope comparison images for **d_mic_**≥10µm, indicating a resolution of ∼10µm. **d_CS_** refers to the center-to-center distances of these resolved fluorescent particles in the decoded lensfree images. For **d_mic_** = 6µm case, however, compressive decoding does not succeed in resolving the particles.

**Figure 3:**
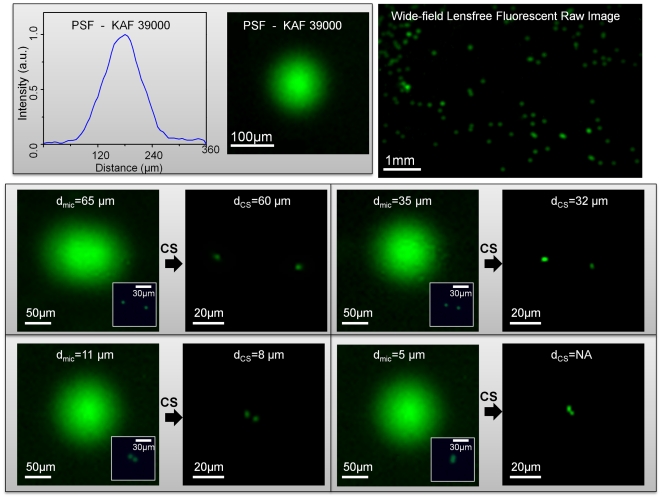
Decoding performance of our lensfree fluorescent imaging platform with a different sensor chip. Same as in Fig. 2, except for KAF-39000 sensor-chip. Similar to Fig. 2, compressive decoding enables a lensfree spatial resolution of ∼10 µm on a chip. Because of its different sensor design, the measured PSF of KAF-39000 is quite different than the PSF of KAF-8300 shown in Fig. 2. This, however, does ***not*** pose a limitation for achieving a similar spatial resolution. These experimental results, together with Fig. 2, successfully demonstrate the sensor-chip independent decoding performance of our lensfree fluorescent imaging platform.

For this end, we first measured the fluorescent PSF of our lensfree platform by imaging isolated 4µm fluorescent beads using these two different CCD chips. Figs. 2 and 3 illustrate these measured point-spread-functions for KODAK KAF-8300 and KODAK KAF-39000 CCD chips, respectively. As demonstrated in these figures, the PSF of each sensor, under similar imaging conditions, is quite different from the other, which is mostly dictated by the opto-electronic design of each CCD chip. For instance, the full-width-half-maximum (FWHM) of KAF-8300 PSF is ∼80µm, which implies a fairly limited resolving power for raw fluorescent images as illustrated in Fig. 2. The same conclusion also applies to KAF-39000 PSF with an FWHM of ∼120µm, as a result of which, closely packed 4µm fluorescent particles cannot be resolved from each other in raw lensfree images (see Fig. 3).

On the other hand, compressive decoding of these raw lensfree images (using the measured PSFs) permits *close to an order-of-magnitude increase* in our resolving power by rapid digital reconstruction of the fluorescent distribution at the object plane (for further details refer to the Experimental Methods Section). The performance of this compressive decoding approach is quantified in Figs. 2 and 3 (for KAF-8300 and KAF-39000 chips, respectively), which both indicate a resolution of ∼10 µm that is independently confirmed using conventional fluorescent microscope images of the same 4µm particle pairs (refer to the inset images in Figs. 2, 3). These experimental results successfully demonstrate the sensor-chip independent decoding performance of our lensfree fluorescent imaging platform.

The resolution limit in our lensfree imaging results is mainly dictated by the detection signal-to-noise-ratio (SNR), since the tails of the measured PSF, after a certain signal strength, fall below the noise floor of the sensor. In these reported experiments (Figs. 2, 3) the CCD chips were kept in room temperature, and therefore further improvement in resolution (beyond ∼10µm) can potentially be achieved by active cooling of the opto-electronic sensors without a trade-off in the imaging FOV, which spans the entire active area of the CCD, i.e., ∼2.4 cm^2^ for KAF-8300 and ∼18 cm^2^ for KAF-39000 (see Fig. 1). We should also note that, with larger area sensors, the imaging FOV of this platform can be even further increased while maintaining a similar resolution level.

On a related note, it is important to emphasize that the pixel size in lensfree compressive imaging is “not” a fundamental limitation for spatial resolution if the detection SNR is sufficiently high. Consider for instance lensfree imaging of two fluorescent points that are directly located on a single pixel. Under this condition, it is theoretically and practically impossible to resolve these two fluorescent points that fall within a single dummy pixel. However, the same two sub-pixel fluorescent points can be resolved from each other using lensfree compressive imaging if several pixels could detect weighted averages of their fluorescent emission. Therefore, under an appropriate detection SNR, if the physical gap between the fluorescent objects and the sensor plane can be increased to perform efficient spatial encoding of the fluorescent objects, resolving of arbitrarily sub-pixel point sources would be feasible. The fundamental limitation to this resolving power is therefore the detection SNR, which determines how many pixels can independently and accurately measure the lensfree fluorescent contributions of the particles. Therefore, for a practical SNR level, there is always an optimum gap range between the object and sensor planes, which we found to be ∼50–200 µm for our CCD chips at room temperature.

**Figure 4:**
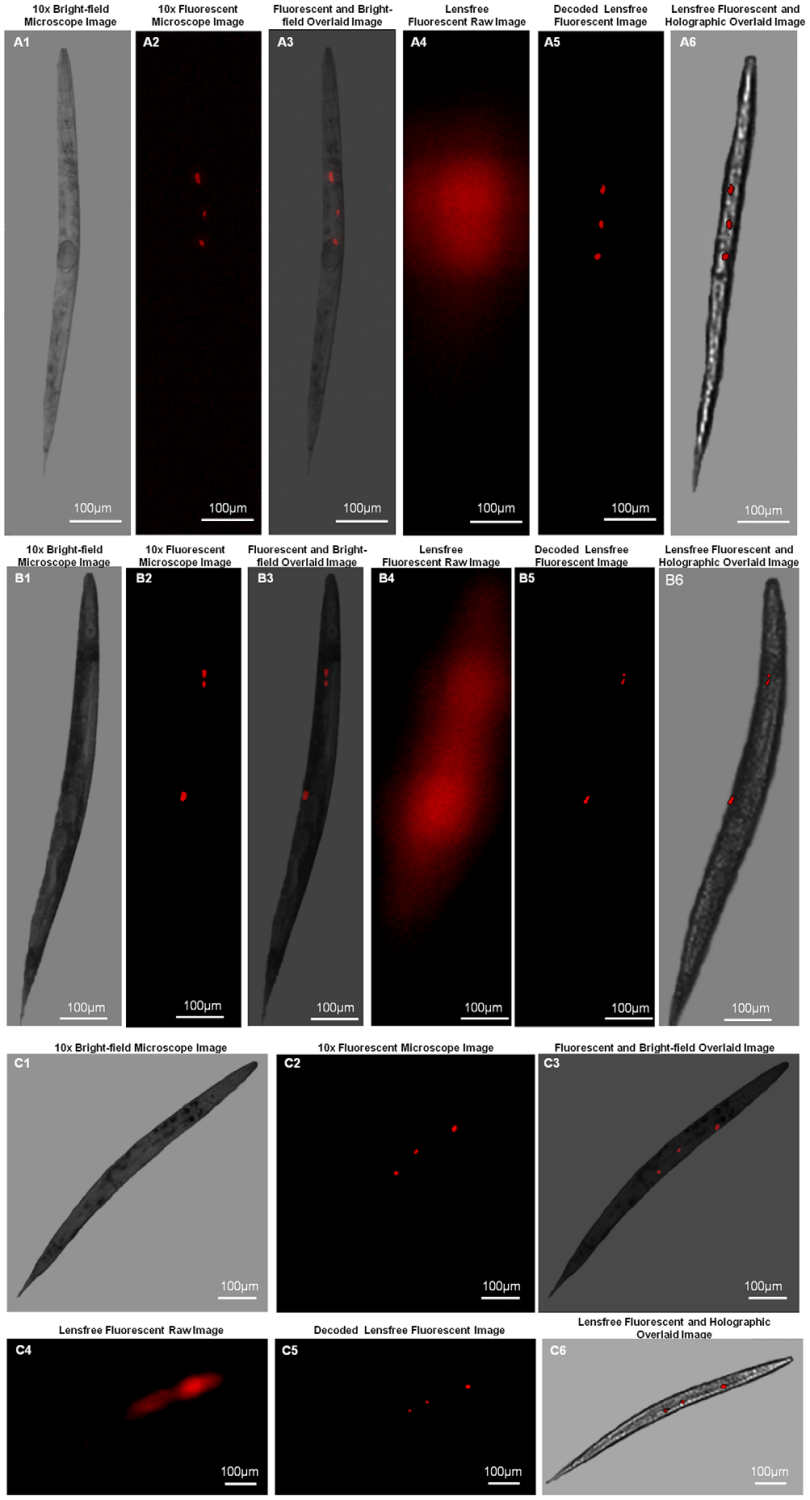
Lensfree fluorescent and holographic transmission imaging of *C. elegans*. Lensfree on-chip fluorescent imaging of transgenic *C. elegans* is shown for three individual animals using KAF-8300 sensor. (a4), (b4) and (c4) illustrate the lensfree fluorescent raw images that all look blurry at the detector plane. Compressive decoding of these blurry patterns enabled digital reconstruction of much higher resolution fluorescent images of these *C. elegans* samples as shown in (a5), (b5) and (c5), respectively. 10× objective-lens fluorescent microscope images of the same worms shown in (a2), (b2) and (c2) agree well with our decoded lensfree fluorescent images. In addition to fluorescent imaging, the same lensfree platform also permits holographic [18] transmission imaging of the same samples such that hybrid images can be created by superimposing the decoded lensfree fluorescent images and the reconstructed holographic images as shown in (a6), (b6) and (c6). Microscope comparisons of the same samples are also provided in (a3), (b3) and (c3), respectively. Slight rotations of the worms are observed between the lensfree decoded images and their microscope comparison images since they are acquired at different experiments.

**Figure 5:**
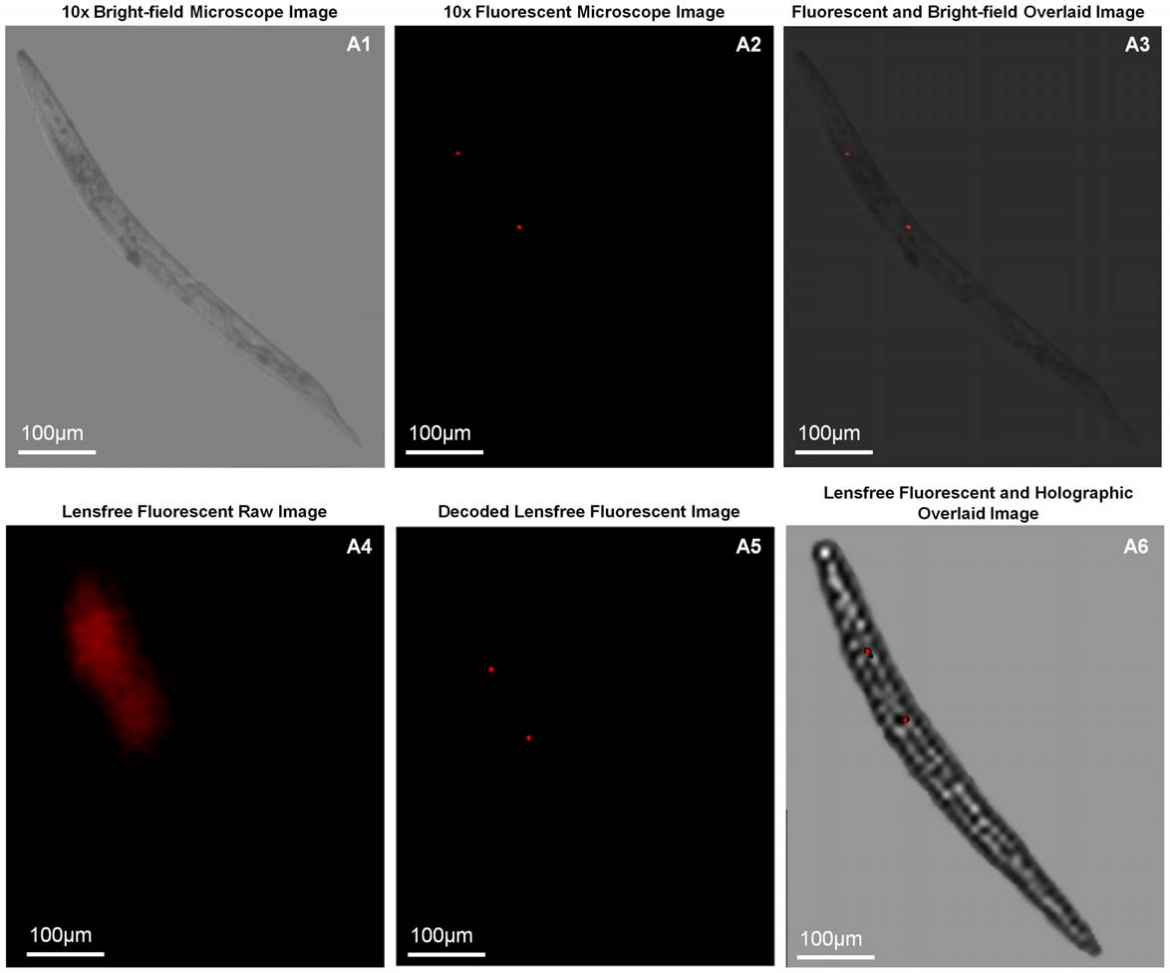
Lensfree imaging of transgenic *C. elegans* with a different sensor chip. Same as in Fig. 4, except this time for a different sensor chip (KAF-11002; 9µm pixel size, 11 MPixel). Similar to Fig. 4, the decoded lensfree fluorescent image of the transgenic *C. elegans* sample provides a decent match to a conventional fluorescent microscope image of the same worm (acquired with a 10× objective-lens, NA = 0.25). Slight rotation of the worm is observed between the lensfree decoded image and its microscope comparison image since the two are acquired at different experiments.

After this initial characterization of the performance of our wide-field fluorescent imaging platform, we next imaged transgenic *C. elegans* samples (refer to the Methods Section for details) over a wide FOV using the same lensfree configuration depicted in Fig. 1. The results of these imaging experiments are summarized in Figs. 4 and 5 (as well as Appendix S1, see e.g., Figures S3 and S4), which also provide conventional fluorescent microscope images of the same samples for comparison purposes. As shown in these figures, raw lensfree fluorescent signatures of the worms are highly blurred due to our broad PSFs. However, using the measured PSF of each platform, these lensfree signatures can be compressively decoded to digitally yield much higher resolution images of the fluorescent regions located within the *C. elegans* body, which very well agree with the images obtained using a regular lens-based fluorescent microscope (see Figs. 4, 5). These experimental results successfully demonstrate the efficacy of our compressive decoding approach to image transgenic *C. elegans* samples using lensfree fluorescent on-chip imaging over an ultra-wide FOV that covers the entire active area of the CCD chip (e.g., >2–8 cm^2^).

We should also note that, the presented on-chip microscopy platform could potentially achieve multi-color imaging of biological samples labeled with multiple distinct targets. In our reported experiments, monochrome CCD chips were used to achieve single color lensfree fluorescent imaging; however, the use of e.g., RGB CCD chips could be utilized to image multiple colors. Unlike conventional lens-based fluorescent microscopy the use of an RGB sensor chip does not immediately bring color imaging capability since without the use of any lenses, all the colors mix with each other at the sensor plane due to unavoidable diffraction. Therefore, lensfree fluorescent imaging might require a more sophisticated compressive decoder to enable separation of multiple colors using raw format RGB images, which was not at the focus of this work.

In addition to fluorescent imaging, our lensfree on-chip platform also permits holographic transmission imaging [18], [19] of the worms using the top interface of the same prism that is used in fluorescent excitation (see Fig. 1). In this lensfree holographic imaging approach, a spatially incoherent quasi-monochromatic source such as a light-emitting-diode (LED) illuminates the samples of interest after being spatially filtered by a large aperture (e.g., 0.05–0.1 mm diameter). This incoherent light source picks up partial spatial coherence that is sufficiently large to record lensfree in-line holograms of the worms on the CCD chip. These acquired in-line holograms can then be rapidly processed using iterative recovery algorithms [18], [19] to create lensfree transmission images of the *C. elegans* samples over the entire active area of the sensor-chip, matching the imaging FOV of the fluorescent channel. Figs. 4, 5 illustrate such reconstructed lensfree holographic images of the samples, where the lensfree fluorescent images of the same worms were also digitally super-imposed, creating a hybrid image of the *C. elegans* (i.e., both fluorescent and transmission). It is evident from these lensfree images that the spatial resolution of our platform is modest compared to a regular lens-based microscope. On the other hand, the main advantages of our platform are its ultra-wide FOV and compact on-chip interface (see Fig. 1) which might provide an important match for ultra-high throughput screening of *C. elegans* samples within automated micro-fluidic systems.

Finally, we would like to also point to an alternative lensfree imaging configuration that can also perform fluorescent imaging of *C. elegans* samples on a chip. In this modified configuration (refer to Appendix S1 and Figures S1, S2 for details), we make use of a fiber-optic faceplate inserted underneath the sample substrate to control and tailor the fluorescent PSF of the imaging platform. Compressive decoding of transgenic *C. elegans* samples using these altered fluorescent PSFs yields similar imaging results as in Figs. 4, 5 (see Appendix S1 and Figures S3, S4). This modified configuration can conveniently tailor the fluorescent PSF of the imaging platform to enhance the detection SNR, especially at larger gaps between the object and sensor planes. This could be an important advantage if physical separation between the sample and the sensor-chip is required. Despite this important flexibility, this faceplate based lensfree imaging approach has one limitation: The holographic imaging channel is now significantly distorted since the modes of the fiber-optic faceplate mess the complex spatial frequency content of the holographic field propagating toward the sensor-array. For further details on this modified lensfree on-chip configuration and its *C. elegans* imaging results, refer to Appendix S1.

In conclusion, we have demonstrated lensfree fluorescent imaging of transgenic *C. elegans* over an ultra wide field-of-view of >2–8 cm^2^ with a spatial resolution of ∼10 µm. This is the first time that a lensfree on-chip imaging platform has achieved *fluorescent* imaging of *C. elegans*. We tested the efficacy of this on-chip imaging approach with different types of opto-electronic sensors to achieve a similar resolution level independent of the imaging chip. Furthermore, we demonstrated that this wide FOV lensfree fluorescent imaging platform can also perform bright-field imaging of the same samples using partially-coherent lensfree digital in-line holography. This unique combination permits ultra-wide field dual-mode imaging of *C. elegans* which could provide a useful tool for e.g., high-throughput screening applications.

## Materials and Methods

### Design of the fluorescent and holographic lensfree on-chip imaging system

Our lensfree imaging system utilizes a rhomboid prism to achieve fluorescence excitation through its side facet as shown in Fig. 1. After interacting with the entire body of the worm, pump photons are rejected by TIR occurring at the bottom glass substrate. To create a sufficient dark-field background, the weakly scattered pump photons that do not obey TIR are also rejected by an additional absorption filter (see Fig. 1), as a result of which only the fluorescent emission from the objects is detected by the opto-electronic sensor-array.

Note that unlike conventional lens-based fluorescent microscopy, the use of thin-film interference filters in our platform is not trivial since rejection of pump photons in a lensfree imaging configuration would require deposition of much thicker interference films to block a large angular range of pump photons. This not only increases the cost but also requires the use of considerably thick substrates due to higher stress in the thicker film, which significantly weakens the SNR of the fluorescent PSF, also degrading the achievable resolution. Therefore, we avoided using such interference based fluorescent filters, and instead, fabricated absorption based filters that have dyes coated on ultra-thin glass substrates (∼30 µm).

The fabrication recipe of these thin absorption filters includes dissolving Orasol dyes in a small volume of cyclopentanone and then adding KMPR 1005 Photoresist (∼0.4 g ml^−1^ dye concentration), after which excess dye material was removed using a 0.45µm diameter mechanical filter [31]. This step is followed by spin coating for 20 s at 2000 rpm, baking for 300 s at 100°C, flood exposure at 13 mW/cm^2^ for 35 s, and finally baking for another 120 s at 100°C. Based on this recipe, we fabricated different long pass absorption filters with cut-off wavelengths of 510nm, 540 nm and 600 nm by using various types of Orasol dyes, including Yellow 2RLN, Orange G, and Red BL, respectively. The rejection ratio (∼30–40 dB) of these fabricated absorption filters is sufficiently large to create the necessary dark-field background (together with TIR), making them rather useful in lensfree fluorescent on-chip imaging applications.

Once fabricated, these absorption filters (total thickness ∼40 µm; 10 µm filter+30 µm glass substrate) were placed directly on the top of the active region of the CCD sensor, acting also as a protector layer for the bare sensor surface. An additional disposable ultra-thin glass substrate (∼30 µm thick) was also used between the sample and the absorption filter.

As for the excitation, an incoherent light source was used, which was coupled from a Xenon lamp spectrally tuned to ∼580 nm (with 15 nm bandwidth) through a monochromator (MS260i, Newport). During our experiments, the total power of excitation was kept at ∼1.0–1.5 mW for an FOV of >2 cm^2^.

In addition to lensfree fluorescent imaging, the same on-chip platform shown in Fig. 1 also permits lensfree holographic [18], [19] imaging of the same samples through the top facet of the same prism that is used in fluorescent excitation. This vertical illumination is achieved by an incoherent source (i.e. an LED, 632 nm peak, and ∼20 nm bandwidth) that is *spatially* filtered with a pinhole (∼0.05–0.1 mm) to achieve holographic transmission imaging within the same on-chip platform.

Finally, we would like to also mention that all the sensors that we used in this work were monochrome. To show color images of fluorescent objects, a custom developed pseudocoloring algorithm was used in Matlab to edit the presented images.

### *C. elegans* Sample Preparation

Transgenic *C. elegans* used in this work is widely studied to better understand the connections between muscle cells and related motor neurons [32]. For this end, UNC 122 gene is co-injected into the worms with a phenotypic marker (mCherry; emission wavelength: 610 nm).

For preparation of these transgenic *C. elegans* samples toward on-chip imaging, a small chunk of nematode growth medium (NGM) was extracted from the culturing plate with a sterilized tool. This specimen was dissolved in a paralyzing medium (∼200 µL) that was prepared with 10mM of Levamisole. To detach the worms from the gel medium, the aliquot is gently vortexed and centrifuged. By using a pipette, transgenic worms are then transferred to our substrates for lensfree on-chip imaging.

We used an immobilization reagent, i.e. Levamisole [33], to avoid hazy images, which also enabled us to capture comparison images of the same samples using a conventional fluorescent microscope. Note also that to avoid physical damage to adult worms, mechanical spacers such as non-fluorescent particles (∼50–100 µm diameter) were also used in our imaging experiments.

### Compressive Decoding of Lensfree Fluorescent Images

As illustrated in Figs. 2, 3, 4, 5 and in Appendix S1, compressive decoding enables accurate reconstruction of the fluorescent distribution at the object plane based on the measured PSF of our lensfree imaging platform, achieving a spatial resolution of e.g., ∼10 µm over >2–8cm^2^ FOV. This numerical recipe relies on compressive sampling theory [25]–[27] which presents a new method to reconstruct a sparse signal from its under-sampled representation. Wide-field fluorescent imaging of *C. elegans* samples on a chip by definition brings sparsity to the imaging problem since most of the FOV is already dark (i.e., non-fluorescent). Based on this connection to compressive sampling theory, lensfree raw fluorescent images can be rapidly decoded (using the measured fluorescent PSF) to significantly improve the resolving power of our platform as demonstrated in Figs. 2, 3, 4, 5 and in Appendix S1.

This compressive decoding process can be formalized as an *l_1_*-regularized least square problem [28], such that:
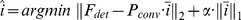
(1)where *F*_det_ is the detected raw fluorescent image at the sensor-array; 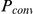 represents the 2D convolution matrix based on the fluorescent PSF of the system; 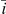 is the fluorescent source distribution that creates the lensfree image at the detector plane; *α* is a non-negative regularization parameter; and 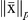 represents the *l_p_* norm of vector 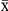. The optimization algorithm used in this work is based on truncated Newton interior-point method [28] which rapidly converges to a sparse fluorescent solution (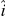) based on Eq. (1) [15].

## Supporting Information

Appendix S1 Use of fiber-optic faceplate in lensfree fluorescent on-chip imaging of transgenic *C. elegans*.
(DOC)

Figure S1 **Use of a fiber-optic faceplate in lensfree on-chip imaging.** An alternative lensfree fluorescent on-chip imaging geometry is shown. The imaging system is kept the same as in Fig. 1, except insertion of the fiber-optic faceplate between the sample and the sensor. This geometry provides SNR advantages especially for operating at large distances between the fluorescent objects and the sensor board.
(TIF)

Figure S2 **Point-spread-function comparison for two different faceplate configurations.** (Top Row) The *measured* PSF of the faceplate (FP) based lensfree imaging geometry shown in Fig. S1 is demonstrated using 10 µm fluorescent particles. The left PSF corresponds to an FP with 0.3 NA, whereas the right one is for an FP with 1.0 NA. Both of these PSFs are significantly narrower when compared to the PSFs reported in Figs. 2 and 3. In (a) and (b), a comparison of the lensfree images of fluorescent micro-particles using these two different faceplates is presented over the same imaging field-of-view. Sensor-chip: KAF-11002.
(TIF)

Figure S3 **Lensfree fluorescent imaging of transgenic *C. elegans* using two different fiber-optic faceplate configurations.** Same as in Figs. 4 and 5, except that the lensfree imaging set-up now involves the use of a faceplate as illustrated in Fig. S1. Our decoded lensfree fluorescent images with both of the faceplates (NA = 0.3 and 1.0) agree well with conventional fluorescent microscope image of the same transgenic *C. elegans*. KAF-11002 sensor-chip was used in these experiments. Slight rotation of the worm is observed between the lensfree decoded image and its corresponding microscope comparison image since the two are acquired at different experiments.
(TIF)

Figure S4 **Lensfree imaging of transgenic *C. elegans* samples with the use of a faceplate on a different sensor chip.** Same as in Fig. S3, except that KAF-8300 sensor-chip was used. Once again the decoded lensfree fluorescent images with the use of a faceplate (NA = 1.0) agree well with conventional fluorescent microscope images of the same transgenic samples. Slight rotations of the worms are observed between the lensfree decoded images and their microscope comparison images since they are acquired at different experiments.
(TIF)
