# Supplementary material for: Lensfree Fluorescent On-Chip Imaging of Transgenic Caenorhabditis elegans Over an Ultra-Wide Field-of-View
Source: PLoS One. 2011 Jan 6;6(1):e15955. doi: 10.1371/journal.pone.0015955 (PMC3017097; doi:10.1371/journal.pone.0015955)
Supplement: Appendix S1 — Use of fiber-optic faceplate in lensfree fluorescent on-chip imaging of transgenic C. elegans. (DOC) [file pone.0015955.s001.doc]

### **Supporting Information - Appendix S1**

Use of fiber-optic faceplate in lensfree fluorescent on-chip imaging of transgenic *C. elegans*

Fiber-optic faceplate is an optical component that is composed of a 2D array of fibers that can relay an arbitrary optical intensity distribution from one side to another. The use of a fiber-optic faceplate in lensfree on-chip imaging (see Fig. S1) can be quite valuable for tailoring the fluorescent PSF of the platform which could also provide an SNR advantage for operating at large vertical distances between the sample and sensor planes. This might especially be useful if physical isolation of the samples from the electronics of the sensor board is desired, i.e., the thickness of the faceplate can be increased without significantly affecting the fluorescent PSF. Therefore for certain applications, the use of such a planar optical component in lensfree fluorescent on-chip imaging could be essential.

Motivated by its potential, we have tested the fluorescent imaging performance of the lensfree set-up shown in Fig. S1 using two different faceplates with numerical apertures (NAs) of 0.3 and 1.0. The thickness of each faceplate was ≥ 3mm which significantly increased the vertical distance between the sample and the sensor-chip, compared to the earlier configuration given in Fig. 1. The comparison of the PSFs of these two faceplate configurations is provided in Fig. S2. The light collection efficiency of the larger NA faceplate is significantly better, since more fibers of the faceplate can guide the fluorescent emission from a point source. This, however, also makes its PSF slightly broader when compared to the PSF of the lower NA faceplate. On the other hand, despite their slight differences, both of the faceplates enable good quality lensfree imaging of transgenic *C. elegans* samples as illustrated in Figs. S3 and S4, where our lensfree compressive decoding results very well match to conventional fluorescent microscope images of the same samples.

Despite its decent fluorescent imaging performance as shown in these results, one limitation of this faceplate based lensfree on-chip imaging approach (Fig. S1) is that unlike the earlier configuration shown in Fig. 1, it does not permit lensfree holographic transmission imaging of the samples from the top. The main reason for this limiting behavior is that the optical modes of the faceplate mess the phase information of the holographic fields that propagate towards the sensor-array. While such fiber-optic faceplates are regularly used for focal-plane arrays to relay intensity images created by a lens-based imaging system, their use in lensfree holographic on-chip imaging would introduce aberrations since the phase information of the holographic fields will now be disordered.

Regardless of this partial limitation, the use of such faceplates in lensfree fluorescent on-chip imaging would be quite useful for high-throughput imaging applications especially when physical isolation of the samples from the electronics of the sensor board is needed.
