# Supplementary figures and images for: Lensfree Fluorescent On-Chip Imaging of Transgenic Caenorhabditis elegans Over an Ultra-Wide Field-of-View
Source: PLoS One. 2011 Jan 6;6(1):e15955. doi: 10.1371/journal.pone.0015955 (PMC3017097; doi:10.1371/journal.pone.0015955)

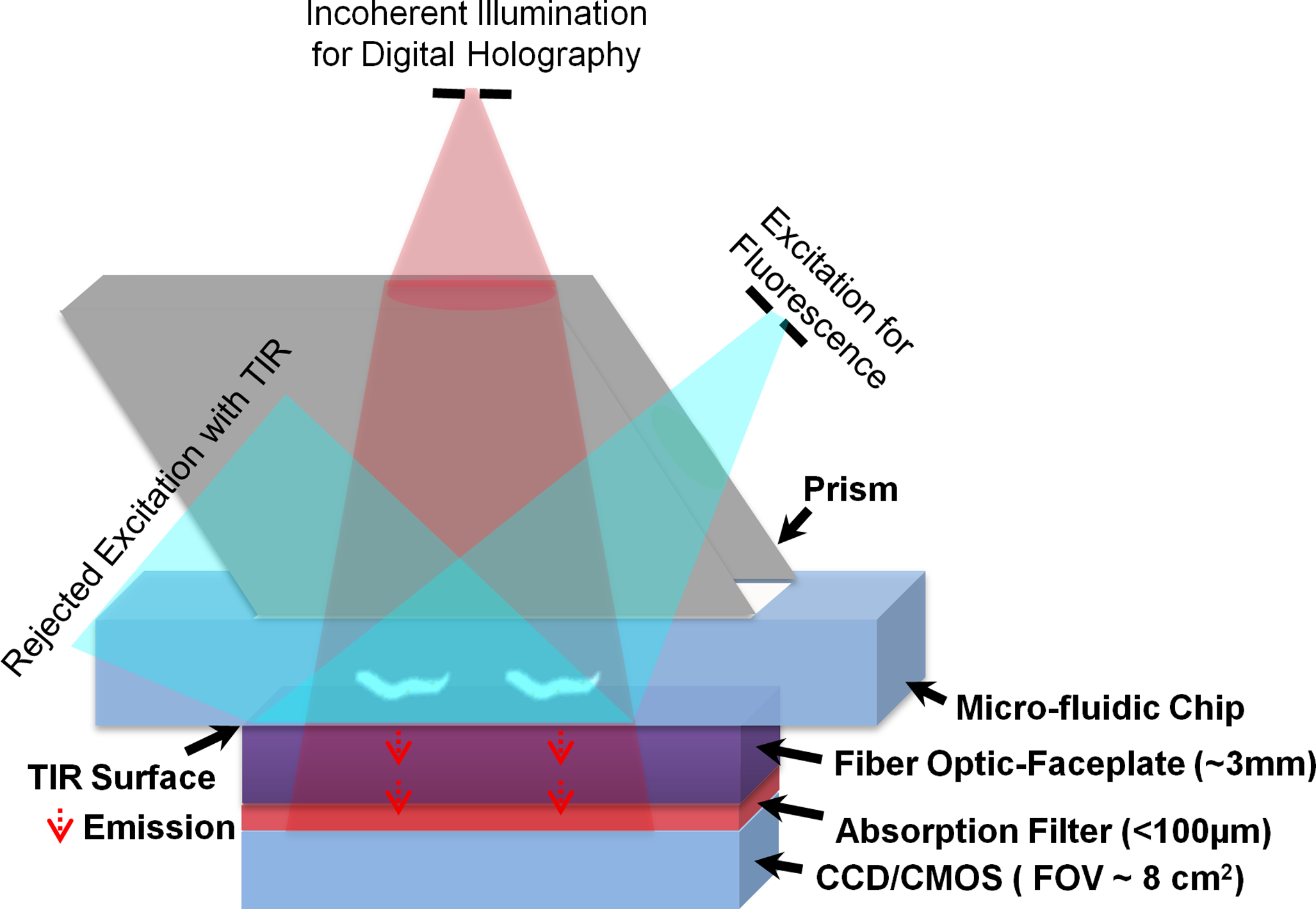

Supplement: Figure S1 — Use of a fiber-optic faceplate in lensfree on-chip imaging. An alternative lensfree fluorescent on-chip imaging geometry is shown. The imaging system is kept the same as in Fig. 1, except insertion of the fiber-optic faceplate between the sample and the sensor. This geometry provides SNR advantages especially for operating at large distances between the fluorescent objects and the sensor board. (TIF) [file pone.0015955.s002.tif]

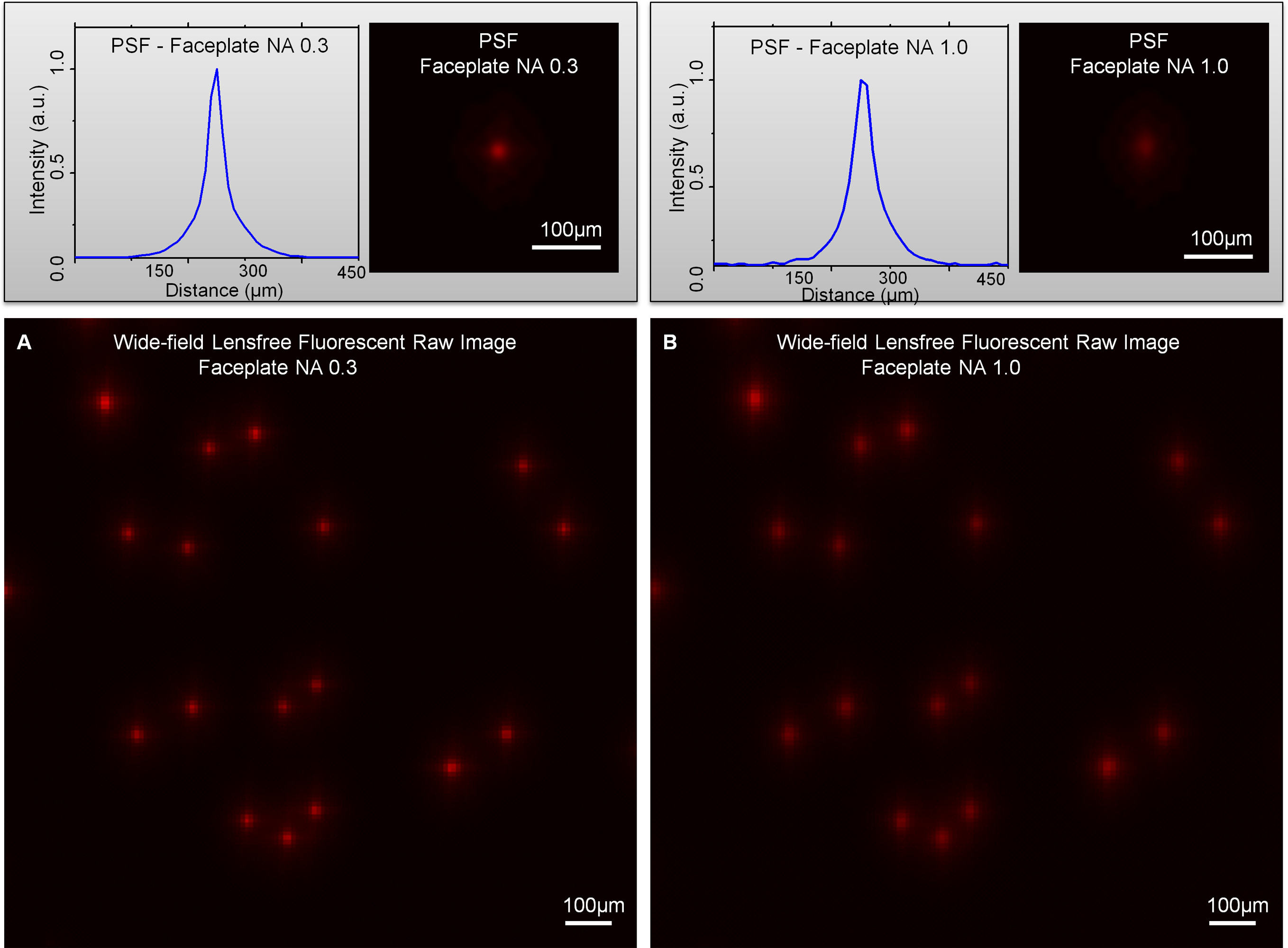

Supplement: Figure S2 — Point-spread-function comparison for two different faceplate configurations. (Top Row) The measured PSF of the faceplate (FP) based lensfree imaging geometry shown in Fig. S1 is demonstrated using 10 µm fluorescent particles. The left PSF corresponds to an FP with 0.3 NA, whereas the right one is for an FP with 1.0 NA. Both of these PSFs are significantly narrower when compared to the PSFs reported in Figs. 2 and 3. In (a) and (b), a comparison of the lensfree images of fluorescent micro-particles using these two different faceplates is presented over the same imaging field-of-view. Sensor-chip: KAF-11002. (TIF) [file pone.0015955.s003.tif]

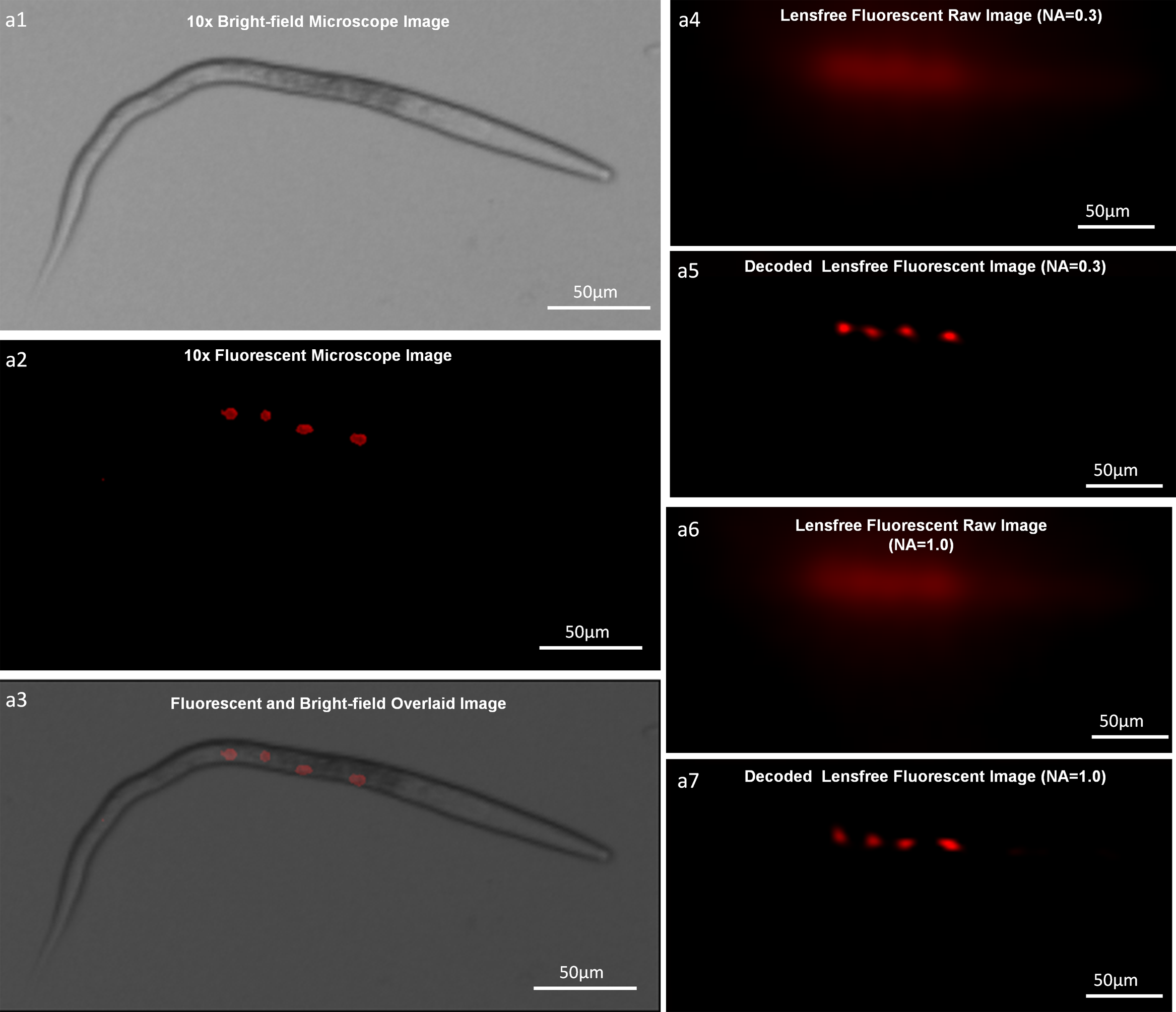

Supplement: Figure S3 — Lensfree fluorescent imaging of transgenic C. elegans using two different fiber-optic faceplate configurations. Same as in Figs. 4 and 5, except that the lensfree imaging set-up now involves the use of a faceplate as illustrated in Fig. S1. Our decoded lensfree fluorescent images with both of the faceplates (NA = 0.3 and 1.0) agree well with conventional fluorescent microscope image of the same transgenic C. elegans. KAF-11002 sensor-chip was used in these experiments. Slight rotation of the worm is observed between the lensfree decoded image and its corresponding microscope comparison image since the two are acquired at different experiments. (TIF) [file pone.0015955.s004.tif]

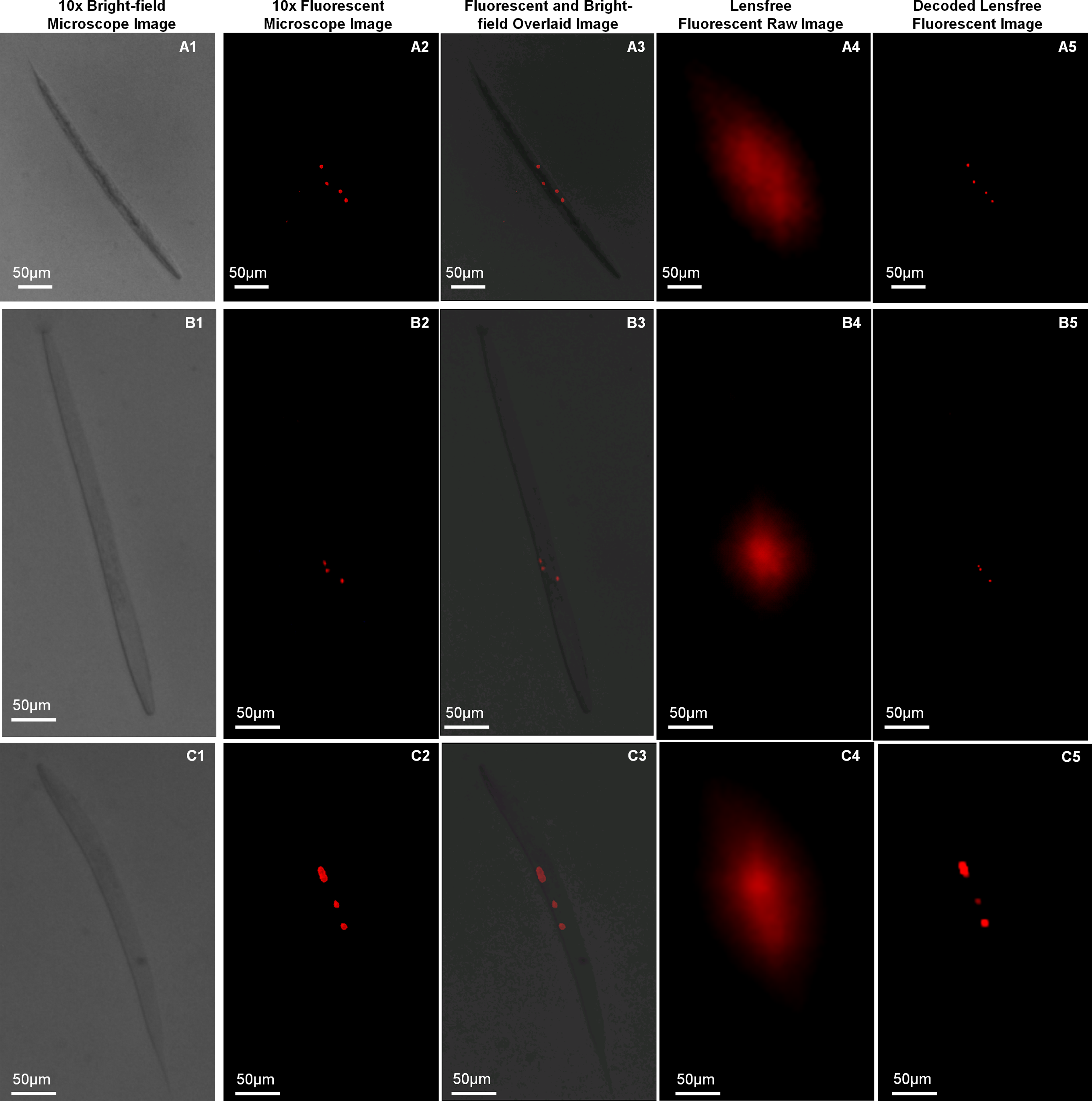

Supplement: Figure S4 — Lensfree imaging of transgenic C. elegans samples with the use of a faceplate on a different sensor chip. Same as in Fig. S3, except that KAF-8300 sensor-chip was used. Once again the decoded lensfree fluorescent images with the use of a faceplate (NA = 1.0) agree well with conventional fluorescent microscope images of the same transgenic samples. Slight rotations of the worms are observed between the lensfree decoded images and their microscope comparison images since they are acquired at different experiments. (TIF) [file pone.0015955.s005.tif]
